# DTI study on rehabilitation of the congenital deafness auditory pathway and speech center by cochlear implantation

**DOI:** 10.1007/s00405-019-05477-7

**Published:** 2019-05-24

**Authors:** Hanlin Wang, Yi Liang, Wenhui Fan, Xia Zhou, Mingming Huang, Guojun Shi, Hui Yu, Guiquan Shen

**Affiliations:** 1 Department of Radiology, General Hospital of the Yangtze River Shipping (Wuhan Brain Hospital), Huiji Road 5#, Hankou, Wuhan, Hubei China; 2 Department of Radiology, The Affiliated Hospital of Guizhou Medical University, Guiyi Street 28#, Guiyang, Guizhou China; 3 Department of Radiology, Guizhou Maternal and Child Health Care Hospital, Ruijin South Road 63#, Guiyang, Guizhou China; 4 Department of ENT, The Affiliated Hospital of Guizhou Medical University, Guiyi Street 28#, Guiyang, Guizhou China

**Keywords:** Congenital sensorineural hearing loss, Cochlear implant, Auditory neural pathway, Speech center, Inferior colliculus

## Abstract

**Purpose:**

To explore the correlation between hearing and speech recovery levels after cochlear implantation and examined the preoperative microstructure of auditory pathways and speech centre using DTI.

**Methods:**

(1) Fifty-two SNHL children between 0 and 6 years and 19 age and gender matched normal hearing subjects had received 3.0 T-MRI examination of the brain.FA, axial diffusion coefficient (*λ*_‖_), radial diffusion coefficient (*λ*_⊥_), and MD values in the lateral lemniscus, inferior colliculus, medial geniculate bodies, auditory radiations, Brodmann areas 41, 42, 22, 44, 45, and 39 were all measured bilaterally. (2) CAP and SIR scores were assessed in fourty-six cochlear implantation children at 6 months post-implant. Correlations among deaf children ages, FA value of bilateral inferior colliculus FA values, BA22, BA44, and postoperative CAP, and SIR scores were analyzed using multiple linear regression.

**Results:**

The preoperative standard partial regression age coefficient of deaf children (|bi′| = 0.404) was slightly greater than that of the inferior colliculus (|bi′| = 0.377) FA value.

**Conclusion:**

Preoperative children ages and inferior colliculus FA values were important factors influencing postoperative CAP score. Inferior colliculus FA value is a vital influencing factor in rehabilitation after cochlear implantation.

## Introduction

Cochlear implantation (CI) can help patients with severe and extremely severe deafness recover partial hearing. It is currently the only effective method to treat children with congenital severe and very severe sensorineural hearing loss (SNHL) [1]. Cochlear implantation is costly and carries some risks. It is currently not possible to realise a detailed pre-operative assessment of a patient’s inner ear structure, auditory language-related central nervous system, and structural brain change, or predict the post-operative hearing and language recovery of children using conventional computed tomography (CT) and magnetic resonance imaging (MRI) [2]. Therefore, we used diffusion tensor imaging (DTI) technology to detect auditory, language-related, brain region microstructural changes in the auditory pathways of congenital sensorineural deaf children. Also, multiple linear regression analysis was performed by combining post-operative follow-up categories of auditory performance (CAP) and speech intelligibility rating (SIR) scores, which provide an important reference basis for pre-operative assessment of cochlear implantation for congenital sensorineural deaf children.

## Materials and methods

### General information

Fifty-two congenital sensorineural deaf children aged 0.9–6 years, who were prepared for cochlear implantation in our hospital from May 2014 to March 2017 were the research subjects. Among the 52 selected subjects, 35 were males and 17 were females, with an average age of 3.32 ± 1.53 years. Inclusion of standards and exclusion criteria: (1) the child, at birth, had no hearing or extremely poor hearing, did not respond to outside general sound. This resulted in extremely severe sensorineural deafness in the pure tone hearing test > 90 dB; (2) After wearing an appropriate hearing aid, hearing and speech training did not achieve any effect after hearing rehabilitation training for 3–6 months. Not included were those with severe inner ear malformation, such as Michel malformation, auditory nerve deficiencies or interruption, serious mental, intellectual, behavioural, or psychological disorders, inability or unwillingness to cooperate with language trainers, and serious mental illness. In contrast, 19 age- and gender-matched children with normal hearing, age 1.17 to 6 years, formed the control group. The control group consisted of 13 males and 6 females, with an average age of 3.8 ± 1.58 years. Temporal bone and head images were normal, and there was no history of neurological disease and family history. The subjects were young and unable to complete the magnetic resonance (MR) examination in an awake state. Subjects were orally administered a 10% chloral hydrate dose of 0.5 ml/kg before the MR examination. Prior to the test, the guardians of all subjects provided signed informed consent. This study was approved by the Hospital Ethics Committee.

### MRI equipment and data acquisition

Cranial axial DTI scanning using a Philips Achieva 3.0T X-Series MR imager in single-shot planar echo imaging sequence (SS-EPI) with a head SENSE eight-channel phased-array coil was performed. The settings were: TR = 5090 ms, TE = 70 ms, layer thickness = 3 mm, layer spacing = 0, FOV = 220 × 220 mm, matrix = 160 × 160. Other conditions were as follows: excitation times = 1; scan layers = 45; b values = 0 mm^2^/s and 800 mm^2^/s; proliferation sensitive gradient direction = 32; scan time = 9:40.

### Analysis

1. Data analysis and processing: a dcm2nii software of MRIcron converted the collected original DICOM 3.0 format files into NIfTI format files. The processed data were registered to b_0_ images using FSL5.0 software. Eddy current correction and realignment were implemented. A fitting tensor model with the least square calculated the subjects’ FA, *λ*_‖_, *λ*_⊥_ and MD numerical map.
2. Extraction of region of interest (ROI): double inferior colliculus, medial geniculate body, BA41, BA42, BA22, BA44, BA45, and BA39 were extracted from a Brodmann template, which was included in PickAtlas software of Wake Forest University (WFU), as a mask. Lateral lemniscus and auditory radiation in a Brodmann template could not be extracted as a mask in this study. The average FA map of the three groups’ subjects after registration was used as a template to sketch lateral lemniscus and auditory radiation information areas. These were added to the Brodmann template (Fig. 1). A mask of the ten information areas was registered to pretreated numerical figures for FA, *λ*_‖_, *λ*_⊥_ and MD for each subject. The ImCalc module of SPM8 software was run on a Matlab platform to calculate average FA, *λ*_‖_, *λ*_⊥_ and MD values for each subject.

**Fig. 1:**
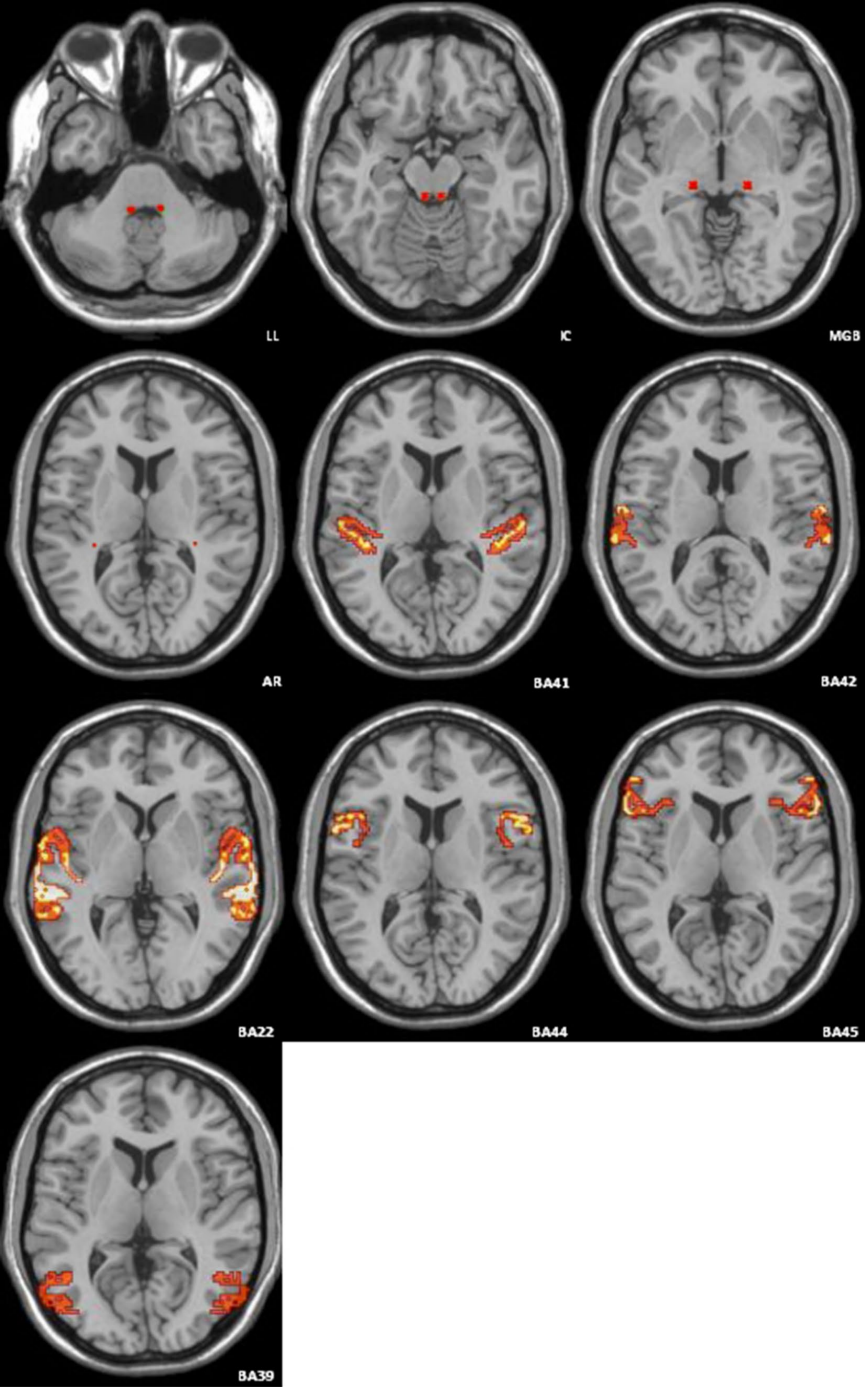
Location of ten regions of interest in the image
3. Post-operative CI follow-up: using an auditory behaviour classification standard proposed by Nottingham University [3], a professional auditory appraiser from the Otorhinolaryngology Hearing Centre of our hospital gave the categories for auditory performance and SIR for 46 CI deaf children.
4. Statistical analysis: multiple linear regression analyses of CAP scores, SIR scores, pre-operative ages, inferior colliculus FA values, and BA22 and BA44 for deaf children 6 months after CI were obtained using SPSS 19.0 software. The difference was considered statistically significant if *p* < 0.05.

## Results

1. ROI-based analysis of deafness group and control group: the fractional anisotropy (FA), axial diffusion coefficient (*λ*_‖_), radial diffusion coefficient (*λ*_⊥_) and mean diffusion (MD) for each subject’s lateral lemniscus, inferior colliculus, medial geniculate body, auditory radiation, BA41, BA42, BA22, BA44, BA45 and BA39 were obtained (Table 1). At the same time, we plotted the auditory pathways and the language central FA values, *λ*_‖_ values, *λ*_⊥_ values and MD value histogram for the deafness and control groups (Fig. 2).

**Table 1:** 0–6-year comparisons of auditory pathway and speech centre DTI-parameters between SNHL patients and healthy controls

	SNHL (SEM)				Control (SEM)			
	FA	*λ*_‖_	*λ*_⊥_	MD	FA	*λ*_‖_	*λ*_⊥_	MD
LL	0.374 ± 0.050	1.394 ± 0.132*	0.764 ± 0.076	0.974 ± 0.081*	0.395 ± 0.042	1.488 ± 0.207	0.803 ± 0.095	1.032 ± 0.121
IC	0.391 ± 0.031*	1.240 ± 0.139	0.659 ± 0.090	0.853 ± 0.103	0.422 ± 0.027	1.315 ± 0.151	0.654 ± 0.070	0.874 ± 0.094
MGB	0.362 ± 0.026	1.175 ± 0.061	0.668 ± 0.052	0.837 ± 0.052	0.355 ± 0.028	1.164 ± 0.043	0.669 ± 0.037	0.834 ± 0.034
AR	0.298 ± 0.049	1.152 ± 0.060	0.722 ± 0.060	0.865 ± 0.049	0.309 ± 0.052	1.168 ± 0.062	0.718 ± 0.048	0.868 ± 0.034
BA41	0.179 ± 0.012	1.047 ± 0.043	0.807 ± 0.045	0.887 ± 0.044	0.184 ± 0.016	1.059 ± 0.049	0.809 ± 0.048	0.892 ± 0.048
BA42	0.093 ± 0.009	1.009 ± 0.064	0.873 ± 0.060	0.919 ± 0.061	0.095 ± 0.008	1.039 ± 0.099	0.886 ± 0.094	0.938 ± 0.096
BA22	0.105 ± 0.007*	1.071 ± 0.057	0.861 ± 0.056	0.913 ± 0.056	0.112 ± 0.011	1.033 ± 0.075	0.866 ± 0.070	0.922 ± 0.072
BA44	0.111 ± 0.008*	1.034 ± 0.078	0.872 ± 0.076	0.927 ± 0.076	0.118 ± 0.008	1.059 ± 0.077	0.886 ± 0.069	0.943 ± 0.071
BA45	0.084 ± 0.008	0.924 ± 0.072	0.794 ± 0.076	0.838 ± 0.074	0.088 ± 0.015	0.955 ± 0.094	0.817 ± 0.087	0.864 ± 0.088
BA39	0.126 ± 0.010	0.876 ± 0.034*	0.708 ± 0.036	0.767 ± 0.034	0.129 ± 0.010	0.899 ± 0.041	0.715 ± 0.032	0.779 ± 0.032

*λ*_‖_, *λ*_⊥_ and MD value units: 10^−3^mm^2^/s

* Statistically significant difference compared to control (*p* < 0.05 Student's t test)

**Fig. 2:**
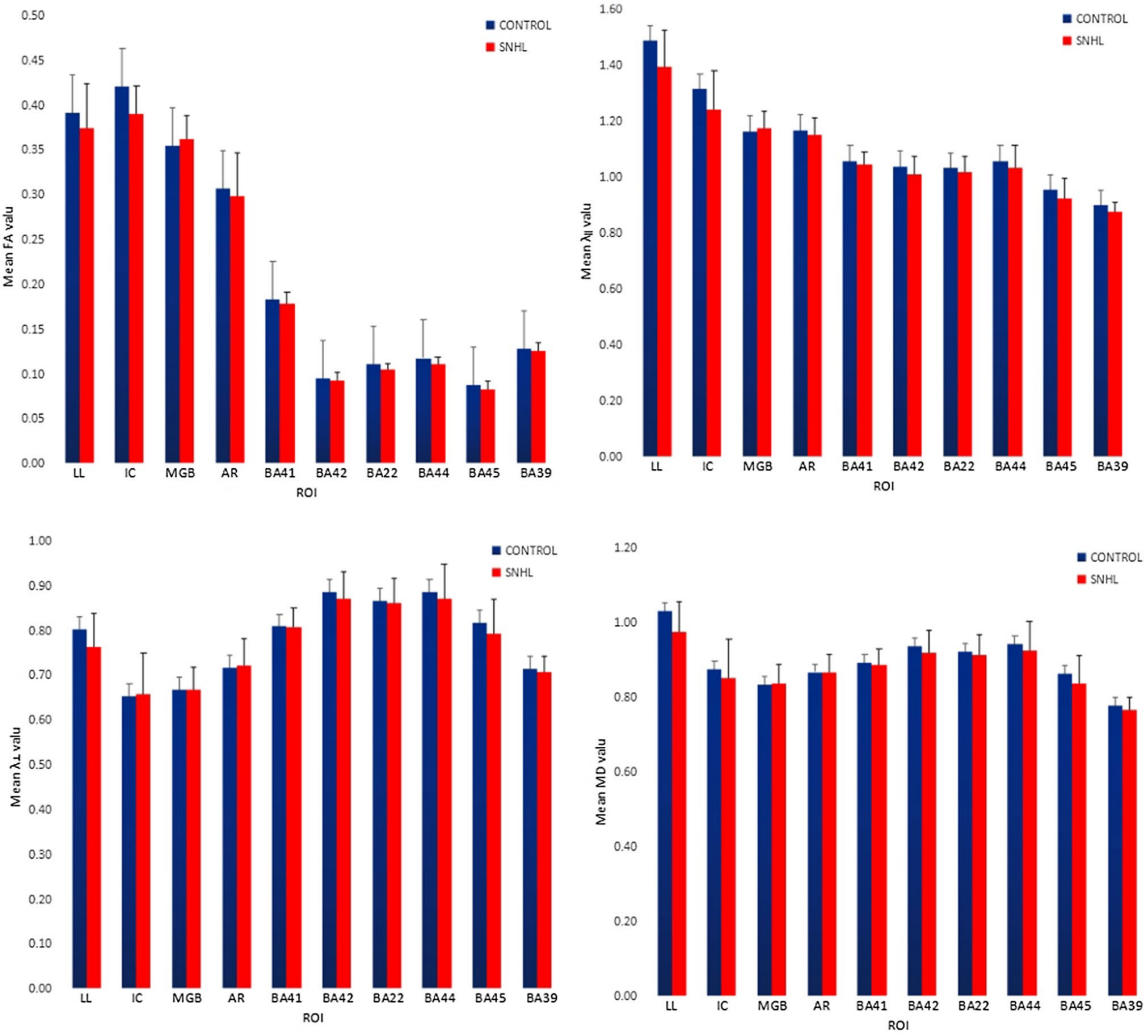
Histogram comparing the auditory pathway and speech centre FA value, *λ*_‖_ value, *λ*_⊥_ value and MD value between SNHL patients and healthy controls aged 0 to 6 years
2. CAP- and SIR-based multiple linear regression analysis: CAP and SIR scores were assigned by auditory appraisers to the 46 SNHL children. A multiple linear regression analysis was performed on the CAP and SIR scores after the CI operation using dependent variables (*y*), and age (*x*_1_), inferior colliculus FA value (*x*_2_), BA22 FA value (*x*_3_) and BA44 FA values (*x*_4_) as corresponding independent variables. The CAP multiple linear regression analysis results of 46 SNHL children appear in Table 2.

**Table 2:** 46 SNHL deaf after cochlear implantation CAP estimation of partial regression coefficient and test

Variable	*B*	Standard error	Beta	*t* value	*p* value
Constant	0.302	2.029		0.149	0.882
*x*_1_	− 0.196	0.074	− 0.405	− 2.644	0.012
*x*_2_	9.042	3.164	0.375	2.858	0.007
*x*_*3*_	− 5.924	17.852	− 0.056	− 0.332	0.742
*x*_4_	5.450	14.400	0.063	0.378	0.707

The multiple linear regression fitting equation *y* = 0.302 – 0.196*x*_1_ + 9.042*x*_2_ – 5.924*x*_3_ + 5.45*x*_4_ was established. The equation hypothesis was further checked. The overall test result was *F* = 4.384 and *p* = 0.005. After removing *x*_3_ and *x*_4_, the results of stepwise regression analysis of partial regression coefficients are shown in Table 3.

**Table 3:** 46 SNHL deaf after cochlear implantation CAP stepwise regression estimation of partial regression coefficient and test results

Variable	*B*	Standard error	Beta	*t* value	*p* value
Constant	0.267	1.218		0.219	0.827
*x*_1_	− 0.195	0.062	− 0.404	− 2.644	0.003
*x*_2_	9.067	3.088	0.377	2.858	0.005

The following stepwise regression equation of multiple linear regression was established: *y* = 0.267 – 0.195*x*_1_ + 9.067*x*_2_.

SIR multiple linear regression for 46 SNHL children was established to further verify the equation hypothesis: *y* = 0.539 – 0.028*x*_1_ + 4.498*x*_2_ + 13.175*x*_3_ –17.635*x*_4_. The overall test result was *F* = 1.435 and *p* = 0.24. The above equation was not statistically significant.

Post-operative CAP multiple linear regression analysis of 46 SNHL children was determined. A standard partial regression coefficient for age was |bi′| = 0.404. A standard partial regression coefficient for inferior colliculus FA was |bi′| = 0.377.

## Discussion

Congenital deafness (CD) is no hearing, or very poor hearing, at birth. CD neonates had no response to general public voice. Most were diagnosed as SNHL. CD incidence among neonates was approximately 1–3% [4]. Saksena et al. found that 50% of CD is attributable to genetic factors, 25% to environmental factors and 25% to unknown reasons [5]. For hereditary deaf patients, extensive research has shown that the most common genes related to deafness include SLC26A4, mitochondrial 12SrRNA and GJB2 [6–8]. The molecular and cytopathological mechanisms of CD are not yet clear and may relate to a variety of molecular, physiological and biochemical changes, such as DNA damage, decreased line body function, changes in intracellular fluid and decreased vascular function.

The human auditory conduction pathway consists of a sensory part and a neuro part. The neuro part involves multiple neuroanatomical structures, including the vestibular cochlear nerve, cochlear nerve nucleus, trapezoid body, superior olivary nucleus, lateral lemniscus, inferior colliculus, medial geniculate body and an auditory centre [9]. A speech centre includes the following: auditory language, motor speech, visual language, writing centre and application centres. The speech composition centre can be described as follows: (1) the auditory language centre includes the primary auditory cortex, the secondary auditory cortex, and the auditory cortex area, corresponding to Brodmann's area (BA) 41, 42, and 22, respectively; (2) the motor speech centre (Broca's area) is located in the posterior gyrus frontalis inferior (mainly on the left and in the dominant hemisphere), BA44 and BA45; (3) the visual language centre is located in the angular convolution of the inferior parietal lobule, BA39; (4) the writing centre is located in the posterior middle frontal gyrus, BA6 and BA8; (5) the application centre is located in the supramarginal gyrus of the inferior parietal lobule, BA40. Based on the above anatomic characteristics of the auditory pathway and motor speech centre, the subjects’ lateral lemniscus, inferior colliculus, medial geniculate body, hearing radiation, BA41, BA42, BA22, BA44, BA45 and BA39 were selected as ROI for the quantitative study.

At present, diffusion tensor imaging (DTI) is the only non-invasive detection method capable of showing the microstructure and pathological status of white matter fibre tracts in vivo [10]. Recently, DTI has been widely used to study congenital sensorineural hearing loss [11, 12]. Most scholars have focused on the lateral lemniscus and inferior colliculus of the auditory pathway when studying SNHL [13, 14]. DTI technology is maturing as used in patients with congenital sensorineural hearing loss; the study of patients with congenital sensorineural hearing loss is no longer confined to the auditory pathway. It now includes changes in brain microstructure. In this study, ROI was not manually sketched, but a Brodmann template of Wake Forest University PickAtlas software was used to extract lateral lemniscus, inferior colliculus, medial geniculate body, hearing radiation, BA41, BA42, BA22, BA44, BA45, and BA39 as regions of interest for quantitative analysis. This avoids such shortcomings as strong subjectivity and poor reproducibility in selecting location and ROI size using a manual ROI sketch. This study found that inferior colliculus FA value decreased the BA22 and BA44 between deaf group and control group. No area was found where *λ*_‖_, *λ*_⊥_ and MD value changes were of statistical significance.

Various information areas in the *λ*_‖_ and MD values of the deaf group were lower than that of the control group. Deaf group *λ*_⊥_ values were higher than that of control group (Figs. 1, 2). Lin et al. studied the lemniscus lateralis (LL) and inferior colliculus (IC) of SNHL patients using an ROI-based analytical method and found that compared to the control group, the FA value in LL and IC FA values of SNHL patients were significantly decreased, while *λ*_⊥_ values were significantly increased, and MD value remained unchanged [15]. Xia Shuang et al. studied patients with prelingual sensorineural hearing loss. They found that patients with sensorineural hearing loss beginning at ages 9–12 years had higher right Heschl's gyrus MD values than the control group. Patients with sensorineural hearing loss beginning at ages 19–22 years had significantly higher bilateral Heschl’s gyrus MD scores than did the control group, and the MD values in the auditory cortex gradually increased with deafness duration [16]. These research results are not consistent with prior reports and may be due to age range, number of research subjects, grouping method and data processing methods. More samples are needed for further validation.

Currently, CI is the most effective method to treat congenital sensorineural hearing loss. Surgery restores hearing to a degree. However, there are still a considerable number of patients with unsatisfactory post-operative hearing improvement. Therefore, it is of great clinical significance to further explore the pre-operative effect evaluation of patients after surgery. The cost of cochlear devices causes patients’ families to have high expectations for prompt recovery of auditory speech functions after CI. Each deaf child has a unique disease situation, and CI effects can be influenced by many factors. Before surgery, patients and their families often inquire about the expected extent of post-operative hearing recovery. Surgeons must accurately evaluate a patient’s inner ear, hearing and language-related central structures before surgery. Accurate predictions of post-operative rehabilitation effects are difficult as many factors affect CI. The most significant clinical factors include deafness aetiology, deafness duration, pre-operative hearing aid use duration, average pre-operative residual hearing, pre-operative language training time and age at CI. Auditory pathway and speech centre structures and functions of the auditory pathway have been insufficiently valued. These factors include auditory centre grey matter-to-white matter volume ratios, brain wave spectrum auditory language-related changes, auditory cortex functional reorganisation and auditory pathway and language central DTI change.

Age is the most significant predictor of the post-operative effects of CI for children with CD. Hassanzadeh et al. held that the best time for CI of children with CD is before 5 years of age. Recovery is even better before 3 years old [17]. Ma et al. reported that earlier CI for pre-speech deaf children would have better results [18]. Harrison et al. pointed out that age is not the only factor influencing CI [19]. Chang et al. conducted a study on 18 patients with congenital SNHL who underwent CI. They found that the FA values in Broca's area FA values, corpus callosum and auditory pathways were significantly lower than those in patients with excellent post-operative CI. Brain area FA values in deaf patients were significantly positively correlated with the post-operative hearing behaviour of CAP and SIR [20]. Wu et al. measured the FA, MD, NAA, NAA/Cr, Cho and Cho/C values in two ROIs of auditory radiation, and superior temporal gyrus in auditory pathway of 92 SNHL deaf children, but only made a simple bivariate linear correlation analysis with post-operative CAP [21].

This study used DTI to detect auditory pathway microstructure changes and auditory-related brain regions in the cortex of SNHL. Correlation analysis was carried out by combining post-operative follow-up CAP and SIR. Multiple linear regression analysis was used in statistical analysis. The influence on pre-operative deaf children of age, FA value of inferior colliculus, BA22 and BA44 to CAP and SIR scores at 6 months after CI was investigated, respectively. The results showed that pre-operative deaf children's age and inferior colliculus FA values had a great effect on post-operative CAP, while the FA value of BA22 and BA44 had little post-operative CAP effect. Pre-operative deaf children’s age and FA value of inferior colliculus, BA22 and BA44 had little effect on post-operative SIR. In a stepwise regression analysis of age and post-operative inferior colliculus FA values, results showed that the standard partial regression coefficient (|bi'| = 0.404) of pre-operative deaf children’s ages was slightly larger than that of the inferior colliculus FA value (|bi'| = 0.377), indicating that the impact of pre-operative inferior colliculus FA values on post-operative cochlear implantation effect was equivalent to that of deaf children’s age. The reason may be that the inferior colliculus is a third-level neuron of the auditory pathway, where auditory reflex synapses are located, and where many upper and lower fibre bundles of the auditory pathway gather. Figures 1, 2 suggests that the FA value of inferior colliculus is higher than that of the other nine information areas. This confirms that the degree of injury to the anatomical structure of the inferior colliculus has an important effect on post-operative CI results. Lin et al. carried out a DTI study of the lateral lemniscus and inferior colliculus of 37 adult SNHL patients, finding that the lesion degree of inferior colliculus was more severe than that of the lateral lemniscus, and that the FA value decline was closely related with clinical deafness degree [15].

This study has some limitations. First, the follow-up time for this study was only 6 months post-CI. At present, it is thought that auditory and verbal recovery tends to stabilise at 12 months post-CI [22]. Correlation analysis in post-operation follow-up evaluations at 12 months or later should lead to more accurate results. Second, the number of subjects in the normal hearing control group was too small for this study. This may have some impact on the results. A key area of study in the next step is to conduct long-term post-operative follow-up evaluations of deaf children, collect more DTI data from the normal hearing control group and further analyse the effect of other influencing factors on post-operative rehabilitation.
